# Metacontrol and joint action: how shared goals transfer from one task to another?

**DOI:** 10.1007/s00426-020-01443-9

**Published:** 2020-11-23

**Authors:** Roman Liepelt, Markus Raab

**Affiliations:** 1 Department of General Psychology, Faculty of Psychology, FernUniversität in Hagen, Hagen, Germany; 2 Institute of Psychology, German Sport University Cologne, Cologne, Germany; 3 Institute for Psychology, University of Muenster, Münster, Germany; 4 School of Applied Sciences, London South Bank University, London, UK

## Abstract

In most of our daily activities and in team sports, we interact with other individuals and do not act in isolation. Using a social variant of the standard two-choice Simon task, this study aims to test if competitive/cooperative processing modes (i.e., metacontrol states) change the degree of bodily self-other integration between two persons in joint action. In addition, and more exploratory the study tested if this effect depends on a shared group experience with the partner. Two participants shared a visual Simon task, so that each person basically performed complementary parts of the task, which transfers the paradigm into a go/no-go Simon task for each person. Before running this joint Simon task, we set both participants either in a competitive or a cooperative control state by means of a dyadic game, a manipulation aimed at testing possible goal transfer across tasks. We found significant joint Simon effects for participants who were in a competitive state and for participants who were in a cooperative state. The joint Simon effect for participants being in a competitive state was significantly smaller than for participants being in a cooperative state. When experiencing the goal induction together with the partner, the joint Simon effect was significantly decreased as when the induction was performed alone. Both effects (metacontrol state induction and shared experience) seem to be statistically independent of each other. In line with predictions of metacontrol state theory, our study indicated that abstract cognitive goal states can be transferred from one task to another task, able to affect the degree of bodily self-other integration across different task situations.

## Introduction

We often work together with other human beings to reach shared goals trying to improve the team performance. But frequently, we also compete against other people either alone (e.g., in a single tennis match) or together with other people (e.g., in tennis doubles) trying to achieve group goals at the expense of others’ goals. When working in independent groups as in bowling or golf, task characteristics of the group performance can be seen as additive (Steiner, 1972). However, in co-acting (e.g., in paddling) or interacting groups (e.g., in basketball), task characteristics of the group performance are typically seen as conjunctive (Steiner, 1972). The aim of this study is to test if competitive/cooperative processing modes (i.e., metacontrol states) between partners change the degree of bodily self-other integration in co-acting dyadic groups. Sebanz, Bekkering, and Knoblich (2006) defined joint action as any form of social interaction, where two or more people coordinate their behavior in space and time to bring about a change in the environment. To coordinate their behavior, two persons need to form a representation of the other person’s action at some level (Liepelt & Prinz, 2011; Vesper, Butterfill, Knoblich, & Sebanz, 2010). Cognitive science investigated if and to what extent people mentally represent their own and other person’s actions (Dittrich, Dolk, Rothe-Wulf, Klauer, & Prinz, 2013; Dittrich, Rothe, & Klauer, 2012; Dolk, Hommel, Prinz, & Liepelt, 2013; Kiernan, Ray, & Welsh, 2012; Liepelt, Wenke, Fischer, & Prinz, 2011; Sebanz, Knoblich, & Prinz, 2003; Wenke, Atmaca, Holländer, Liepelt, Baess, & Prinz 2011) and other person’s tasks (Klempova & Liepelt, 2016; Sebanz, Knoblich, & Prinz, 2005; Yamaguchi, Wall, & Hommel, 2018, 2019), and how this impacts both persons’ behavior. A prominent paradigm of this line of research is the social or joint Simon paradigm (Sebanz, et al., 2003), in which two people share the standard version of the Simon task (Simon, 1969; Simon, Hinrichs, & Craft, 1970). The classic version of the Simon task is a two-choice task, in which one participant has to decide between two different items of a specific category (e.g., color or form) and to respond by pushing one of two laterally located response keys. This stimulus randomly appears laterally on the left or right side of a computer screen, whereby the stimulus location is task irrelevant. Even though stimulus location is task irrelevant, participants show a stimulus–response (S–R) compatibility effect. This S–R effect typically shows faster reaction times when stimulus and response location correspond (compatible trial), compared to when they do not correspond (incompatible trial). This effect is known as the Simon effect (Simon, et al., 1970; Simon & Small, 1969; Simon & Wolf, 1963). Sebanz et al. (2003) transformed the Simon task into an individual go/no go task, so that the individual has to respond to only one of two possible stimuli, while having to withhold the response to the other stimulus. In their study, Sebanz et al. showed that the Simon effect breaks down when performing this go/no go task alone. In addition, a third condition was added, in which the same Simon task was distributed across two persons, each person doing only half of the task, so that both persons now share the two-choice Simon task. As in the go/no go task condition, each person has to respond to only one of the two stimuli and to withhold the response to the other stimulus, whereby now the co-actor takes over the response to the other stimulus. In this joint-task setting, the Simon effect reemerged, but now across both persons sharing the task. This effect was therefore called the social Simon effect (Sebanz et al., 2003) or joint Simon effect—short the JSE.

## The joint Simon effect as a measure of bodily self-other integration

The standard Simon effect is typically explained by the assumption that the spatial response dimension (left/right key press) overlaps with the spatial stimulus dimension (left/right). Hence, task irrelevant spatial stimulus features prime corresponding response features decreasing reaction times when the task irrelevant stimulus dimension is compatible with the required response. Due to a conflict between the instructed and the primed response on S–R incompatible trials reaction times are increased, as conflict resolution takes time. The individual go/no-go Simon effect is abolished because there is only a single response and hence, no left/right response dimension is present anymore. Along these lines the Simon effect is re-established in the joint go/no-go Simon task condition (JSE), because a salient spatial response dimension is restored by the other individual controlling the other response key (Liepelt, et al., 2011). Therefore, Sebanz et al. (2003) concluded that other’s action is regarded as similar to ones own action and other’s task is regarded as own task, making the JSE a measure of the co-representation of other person’s action (Sebanz, et al., 2005). Based on studies showing that JSE-like compatibility effects are also established when one of the two human co-actors is replaced by an event-producing object (Dolk, et al., 2013; Puffe, Dittrich, & Klauer, 2017; Stenzel & Liepelt, 2016), for instance a puppet (Müller, et al., 2011) or a humanoid robot (Stenzel et al., 2012; Stenzel, Chinellato, del Pobil, Lappe, & Liepelt, 2013), a sole mechanism of action co-representation accounting for the JSE and JSE-like effects has been questioned (Dolk, et al., 2011, 2014; Klempova & Liepelt, 2016). Based on the theory of event coding (Hommel, Müsseler, Aschersleben, & Prinz, 2001), the referential coding account has been proposed for joint action (Dolk, et al., 2014, 2013). The basic assumption is that late stages of perception and early stages of action planning and control are cognitively represented by the same kinds of perceptual codes (Prinz, 1997, 2015). Given a similarity between internally used and externally activated events (e.g., events produced by another responding human co-actor) an action discrimination conflict arises during the shared go/no-go decision (Liepelt, et al., 2011). According to the theory of event coding (Hommel, et al., 2001), action selection refers to the selection of a given event that is associated with the to be performed action from a pool of all activated event representations (Dolk, et al., 2013). When sharing the joint Simon task, perceived (or imagined) events produced by the co-actor possessing a high similarity to those events used to select one’s own action produce an action discrimination conflict. According to the referential coding account (Dolk, et al., 2013), this conflict can be resolved by changing the selection criteria through increasing the task relevance of discriminating task features. In the case of the joint Simon task, one dominant discriminating feature is location information (left–right feature codes). For example, strengthening the right response coding (of the right co-actor) strengthens the spatial response dimension, and hence increase the dimensional overlap with the task-irrelevant spatial stimulus dimension (Kornblum, Hasbroucq, & Osman, 1990), producing the JSE. Higher perceptual or conceptual similarity between both the co-actors or their given responses may strengthen the discrimination problem and hence the JSE (Dolk & Prinz, 2016).

Even though different accounts for the JSE were proposed, almost all available accounts do agree that the JSE can be seen as a measure of bodily self-other integration (Iani, Anelli, Nicoletti, Arcuri, & Rubinchi, 2011; Tomasello, Carpenter, Call, Behne, & Moll, 2005). The degree of self-other integration seems to depend on the cognitive states of the individuals sharing the task (Colzato, van den Wildenberg, & Hommel, 2013; Colzato, de Bruijn, & Hommel, 2012; Colzato, Zech, Hommel, Verdonschot, van den Wildenberg, & Hsieh, 2012).

Hommel and Wiers (2017) proposed that cognitive states related to persistence or flexibility directly affect action control (for an earlier account of cognitive control state theory see Goschke, 2003). According to Hommel (2015), differences in cognitive states can be characterized by more (or less) top-down influence of the current action goal leading to less (or more) self-other integration, and strong (or weak) mutual competition between alternative action representations (Ma & Hommel, 2018). Having a task goal related to persistence should therefore lead to less self-other integration, while shared task goals related to flexibility should lead to more self-other integration. The present study tested if shared goals (competition vs. cooperation) two persons have while sharing one task are transferred to a new joint Simon task. Further, and more exploratory, we tested whether shared group experiences shape a potential goal transfer.

## Competition and cooperation in joint action

The bodily self-other integration process (Colzato, et al., 2013; Liepelt et al., 2012) has been shown to depend on the relationship between both co-actors sharing the task (Hommel, Colzato, & van den Wildenberg, 2009; Iani, et al., 2011; Mendl, Fröber, & Dolk, 2018; Ruissen & de Bruijn, 2016; Ruys & Aarts, 2010). When co-actors were friendly, inviting for cooperation, their actions were more strongly integrated than actions of intimidating and unfriendly co-actors (Hommel, et al., 2009) indicating that self-other integration is sensitive to the social relationship between co-actors. Ruys and Aarts (2010) aimed to investigate the independence between actors sharing the joint Simon task. When only the best subject won a reward, the JSE was decreased as compared to a condition in which the team won a reward (cooperation) and when a random selection of team winners earned a reward (competition). This study suggests that interdependency leads to more self-other integration. Iani et al. (2011) argued that cooperation is directly implied by the positive interdependence as two persons sharing a task need to work together for attainment of a common goal. On the other hand, competition is implied by negative interdependence, as both co-actors work against each other for the attainment of a personal goal (see Iani, et al., 2011, p. 442). Further, they argued that the random winner selection used in the competition condition in the study of Ruys and Aarts (2010) was not satisfying, as competition is typically defined as a condition in which one individual attempts to outperform another individual in a zero-sum situation (Iani, et al., 2011). Therefore, Iani et al. (2011) more directly manipulated cooperation vs. competition by instructing participants that either the best couple would receive an extra reward (to induce cooperation) or that the best participant of a couple would receive an extra reward (to induce competition). When performing the joint Simon task together with a person with whom the reward was shared (cooperation), the JSE was increased as compared to a situation, in which the task was played against another person (Iani, et al., 2011) and only the best person won the reward (competition). Combining this logic with an analysis of the sequential modulation of the JSE (Liepelt, Wenke, & Fischer, 2013; Liepelt, et al., 2011; Yamaguchi, Wall & Hommel, 2017), Mendl et al. (2018) found the difference between cooperation and competition conditions only for actor repetition trials, not for trials in which the actor switched. This suggests that processing adjustments primarily affected the processing of one’s own actions. When competing with another person, both actors seem to focus more on their own action leading to less self-other integration. Ruissen and de Bruijn (2016) decoupled the cooperation-competition manipulation from the joint Simon task by letting participants play Tetris before running the actual Simon task. Couples who cooperatively played Tetris with each other showed an increased JSE as compared to couples who competed in the Tetris game. This finding is in line with studies showing that self-construal priming drawing attention to independence reduced the JSE relative to a task drawing attention to interdependence (Colzato, de Bruijn, et al., 2012; Colzato, Zech, et al., 2012). Evidence for a carry-over effect of competition was shown by Iani, Anelli, Nicoletti and Rubichi (2014). In their study, pairs of participants performed a joint Simon task before and after a joint Flanker task. In their first experiment, the joint Simon task was performed under neutral task instructions, while participants performed the joint Flanker task either under cooperation or competition. In the second experiment, participants were required to compete in the joint Flanker task and to cooperate during the subsequent joint Simon task. While the competition effect transferred from the joint Flanker task to the joint Simon task indicated by an absence of a joint Simon task in their first experiment, prior competition did no longer affect joint Simon task performance when a new cooperation goal was introduced in the joint Simon task (Iani, et al., 2014). Cooperation goals and competition goals seem to affect the performance in a joint Simon task and the findings of Iani et al. (2014) indicate that competition seem to extend beyond a specific setting affecting the following interaction. These manipulations, playing Tetris, self-construal priming, or a competition induction, changed the task goal of both persons who are sharing the task, which may explain observed changes in the degree of bodily self-other integration (Colzato, Zech, et al., 2012).

## The present study

To induce a cooperative goal, we adopted the manipulation of Iani et al. (2011), instructing pairs of participants that the best couple will win an extra reward. During the instruction, we did not specify what participants would receive as the “extra reward”. The winner could choose from a random selection of sweets. This condition was contrasted to a condition in which the best individual of each pair will win, earning an extra reward (same type of reward), thereby setting participants in a competitive task mode. However, in contrast to most previous studies testing the impact of cooperation and competition directly in the joint Simon task (Iani, et al., 2011; Ruys & Aarts, 2010) or between two different compatibility tasks (Iani, et al., 2014) showing evidence for a carry-over effect of competition, we induced competitive or cooperative task goals in a separate dyadic game (Colzato, et al., 2013; Ruissen & de Bruijn, 2016) called “Mindflex”. This was done to set participants either in an abstract higher-level competitive or cooperative cognitive state and test the impact of the respective state on bodily self-other integration in a separate task (i.e. abstract goal transfer). The effect of goal transfer was tested with the joint Simon task. Further, and more exploratory, we manipulated whether goal induction was performed alone or together with the partner (i.e. jointly) testing if a possible goal transfer depends on a shared group experience.

If shared competitive task goals can be transferred from one task to another, we predict less self-other integration when both participants are previously set to a competition goal compared to a cooperation goal (JSE__competition goal_ < JSE__cooperation goal_). More exploratory, we tested if this effect depends on shared group experience, which may predict competition goal transfer to be stronger when participants experience the induction together compared to being alone.

## Method

### Participants

In line with a previous study testing the effect of positive interdependence implying cooperation vs. negative interdependence implying competition on the joint Simon effect (Iani, et al., 2011), we included 32 healthy participants (19 female, *M*_age_ = 23.7 years, SD_age_ = 5.3 years) with normal or corrected-to-normal vision in this study. Using the simulation software PANGEA (Westfall, 2016) to estimate the power assuming a small hypothetical effect size (*d*) of 0.3, 32 replicates, 32 participants, and 4096 total observations, indicated that a planned three-way interaction would be gained with sufficient power of 0.801. All participants were right-handed, naive with regard to the hypotheses of the experiment, were rewarded by course credits, and gave their written informed consent before their inclusion in the study. All the procedures were conducted in accordance with ethical guidelines of the local ethics committee of the University of Muenster and the 1975 Declaration of Helsinki.

### Stimuli and procedure

#### Metacontrol state induction

Metacontrol state induction was accomplished using the Mindflex Duel game (NeuroSky, 2018) enabling participants to either jointly (two persons wearing a headset) or individually (one person wearing a headset) control the movement of a flying ball. Each headset consisted of a forehead sensor and an ear clip. The forehead sensor was placed on the person’s correct head area by locating a colored ring on the strap above the left eye. With the headset participants controlled a puck (fan nozzle) located on the game console. The puck could move along a vertical midline of the console. The ball could be raised higher or lower by changing the amount of attention. Through focusing attention participants controlled the movement of the flying ball into a goal position. The higher the concentration level by focusing all attention on the ball and imagining a picture of the raising ball, the more the ball floated up to a height of 5 in. (maximum). Relaxing the body lowered the ball. The amount of concentration was visually displayed as feedback by a concentration meter made of three lights. The Mindflex game was located on a table in the laboratory and two chairs were placed on the table for the players. For the present experiment, we always used the same type of parcour made of a flip frame, a wind wheel, and a flex tower with three hoops (for more information please see Mindflex Duel game instruction manual).

Before the induction, both players had a short practice of about two minutes where each player could test the Mindflex device. The game could be played in cooperation mode or competition mode under shared experience and individual experience conditions, each condition played for five minutes.

##### Cooperation mode (shared experience)

One player controlled the height of the ball by concentration level (the greater the concentration level, the higher the ball floated) and the other player controlled the forward–backward movement of the ball along the vertical midline by concentration level (high concentration caused the puck to move forward, medium concentration caused the ball to stop, and low concentration caused it to move backwards). Both players were required to coordinate their actions to reach the goal position. The best couple of all couples taking part in the experiment won the extra reward.

##### Competition mode (shared experience)

One player controlled the height of the ball by concentration level (the greater the concentration level, the higher the ball floated) and the forward–backward movement of the ball with a manual button trying to reach the goal position faster than the opponent. The other player also controlled the height of the ball by concentration level (the greater the concentration level, the higher the ball floated) trying to disturb the opponent. Roles were switched. The winning points of each player were counted individually and the best individual of each pair won the extra reward.

##### Cooperation mode (individual experience)

The parcour had to be played individually, but the winning points of both players were counted together. Each individual player controlled the height of the ball by concentration level (the greater the concentration level, the higher the ball floated) and the forward–backward movement of the ball with a manual button playing alone. The best couple of all couples taking part in the experiment won the extra reward.

##### Competition mode (individual experience) 

The parcour had to be played individually against each other. Each player tried to reach the goal position faster than the opponent, and the winning points of each individual were counted separately. Each individual player controlled the height of the ball by concentration level (the greater the concentration level, the higher the ball floated) and the forward–backward movement of the ball with a manual button playing alone. The best individual of each pair won the extra reward.

Possible transfer effects of the different inductions were tested in a consecutive joint Simon task.

### Manipulation check

As a manipulation check for the induction of competitive vs. cooperative states via the Mindflex game, we measured three different rating scores under competition and cooperation. 1. Perceived cooperation rating: how cooperative did you feel during the Mindflex task? This rating consisted of an 11-level Likert scale ranging from − 5: very competitive, 0: neutral anchor midpoint, to + 5: very cooperative. 2. Perceived team/group rating: how much did you feel as a group/team with the other person during the Mindflex task? This rating consisted of a 5-level Likert scale ranging from 0: not at all as a group, 2: moderately (midpoint), to 4: completely as a group. 3. Perceived task sharing rating: did you feel that you were acting together with your partner during the Mindflex task? This rating consisted of a 5-level Likert scale ranging from 0: not at all, 2: moderately (midpoint), to 4: completely.

Previous studies showed that positive mood (Kuhbandner, Pekrun, & Maier, 2010) and positive social relationships (Hommel, et al., 2009) may lead to an increased self-other integration in shared tasks. Besides measuring effects of the metacontrol state induction on bodily self-other integration with the joint Simon task, we also tested if the induced competitive or cooperative state induction may affect the conceptual self-other relation with the including other in the Self (IOS) scale (Aron, Aron, & Smollan, 1992). The scale contained six pictures, each showing two circles that varied in overlap to different degrees ranging from 1 (no overlap) to 6 (near full overlap). Participants rated subjectively what they think represents best the degree of overlap to the co-actor.

To also control for possible effects of mood changes (Hommel, et al., 2009; Kuhbandner, et al., 2010) that may go along with a switch from competitive to cooperative states, we measured mood changes (valence and arousal, each on a nine-point scale) before the experiment (baseline), after the game induction, and after the joint Simon task in two experimental blocks by applying the affect grid (Russel, Weiss, & Mendelsohn, 1989).

### Joint Simon task

The experiment was performed in a sound-attenuated, dimly lit room. The experiment was conducted using the experimental software ERTS, version 3.33e (Beringer, 2000). In the joint Simon task (Sebanz, et al., 2003), either a square or a diamond was randomly presented to the left or to the right side of the screen (Liepelt, et al., 2011; Porcu, Bölling, Lappe, & Liepelt, 2016). Responses were recorded with two response keys that were placed on a table at a fixed distance of 15 cm between both keys (Porcu, et al., 2016). The stimuli were displayed on a computer monitor in white on a black background, at a constant viewing distance of 60 cm. The fixation point in the center of the screen was marked by a plus sign (0.9° × 0.9°). Stimuli consisted of squares and diamonds (1.9° × 1.9°), presented to the left or right of the fixation with an eccentricity of 9.5° visual angle.

Two persons were seated side-by-side and each person responded with a key press whenever the assigned stimulus appeared on the monitor. Stimuli were presented randomly, so that both persons performed a turn-taking task.

One person (left person) of the dyad responded to one of two possible shapes (square) by making a simple discrimination response, while the other person (right person) responded to the other shape (diamond). The left hand of both persons remained on their lap. The sitting position of the participants in the joint Simon task remained identical. Stimulus position was task irrelevant. Responses had to be given as fast and accurately as possible. Compatible and incompatible conditions were determined by the combination of the side of participant’s response (left–right) and the task-irrelevant stimulus position (left–right). For compatible trials, response position and stimulus position spatially corresponded (e.g., left–left), while they did not correspond for incompatible trials (e.g., left–right).

Response times (RTs) and error rates of the key presses were recorded. We adopted the trial timing of Liepelt et al. (2011). Each trial started by displaying a central fixation cross (250 ms). Then one of two possible targets (either a square or a diamond) was displayed together with the fixation cross (150 ms). The response window was 1800 ms. In the case of a correct response, the fixation cross was given as feedback (300 ms). When a wrong response was given, the error feedback (“Fehler”, engl. “Error”) was provided (300 ms). When no response was given within the response window time of 1800 ms, the feedback “zu langsam” (engl. “too slow”) was shown (300 ms). After the feedback, a constant inter-trial interval of 1750 ms was provided (Liepelt, et al., 2011).

Prior to the experimental session, participants performed 16 practice trials, followed by the experimental phase of 256 trials for each dyad split in two blocks.

### Study design

A 2 (compatibility) × 2 (shared experience) × 2 (metacontrol state) mixed factorial design was applied with compatibility and shared experience, both as within-subjects factors and metacontrol state as a between-subjects factor. While compatibility varied randomly within each block, the factor Shared experience^1^ was counterbalanced across pairs of participants.

## Results

### Manipulation check (competition vs. cooperation state induction) and emotional controls

**Fig. 1:**
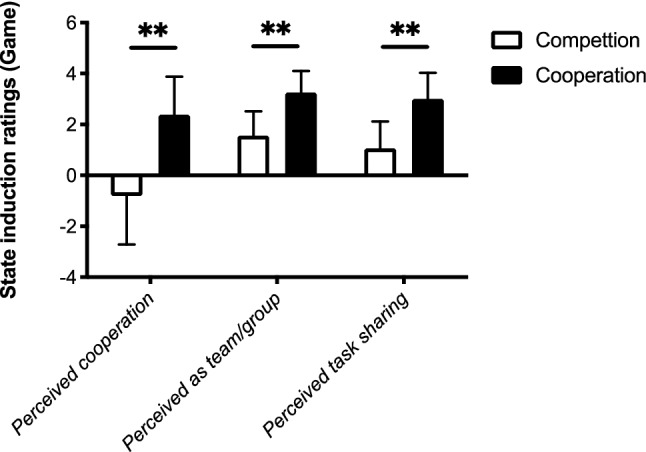
Mean state induction ratings through the Mindflex game and standard deviations (error bars) under competition mode and cooperation mode for perceived cooperation ratings (left), perceived team/group ratings (middle), and perceived task sharing ratings (right).** *p* < 0.001

As a manipulation check to test if the competitive/cooperative state induction was effective via the Mindflex game, we performed a Mann–Whitney *U* test with metacontrol state (competition, cooperation) as a between-subjects factor on the perceived cooperation ratings, perceived team/group ratings, and the perceived task sharing ratings regarding the Mindflex game (see Fig. 1). Descriptive values of the ratings are also displayed.

The perceived cooperation ratings showed a significantly lower rating score after the competition induction (− 0.81) than after the cooperation induction (2.38) (*p* < 0.001). For the perceived team/group ratings, we observed a significant lower rating score after the competition induction (1.56) than after cooperation induction (3.25) (*p* < 0.001). The perceived task sharing ratings indicated a significantly lower rating score after the competition induction (1.06) than after the cooperation induction (3.00) in the Mindflex game (*p* < 0.001).

**Fig. 2:**
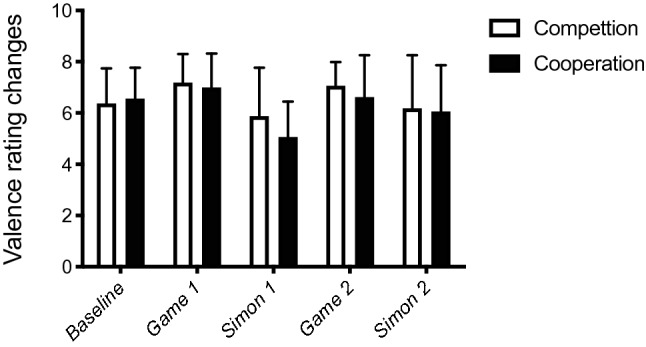
Mean valence rating changes and standard deviations (error bars) under cooperation mode and competition mode at the beginning of the experiment (baseline) and after the first Mindflex game (Game 1), the first Simon block (Simon 1), the second Mindflex game (Game 2), and the second Simon block (Simon 2)

**Fig. 3:**
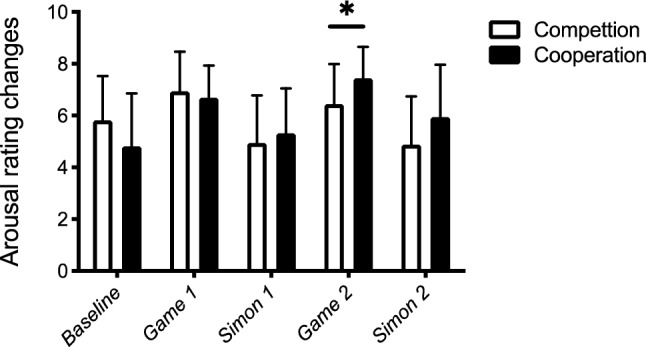
Mean Arousal rating changes and standard deviations (error bars) under cooperation mode and competition mode at the beginning of the experiment (baseline) and after the first Mindflex game (Game 1), the first Simon block (Simon 1), the second Mindflex game (Game 2), and the second Simon block (Simon 2). *p < 0.05

To control for potential emotional changes between competition and cooperation conditions, we submitted the affect grid rating data (Russel, et al., 1989) for valence and arousal to a Mann–Whitney *U* test with Metacontrol state (competition, cooperation) as a between-subjects factor. The valence ratings (see Fig. 2) did not differ between cooperation and competition (all *ps* > 0.18). Arousal ratings (see Fig. 3) did not differ for baseline, Game 1, Simon block 1, and Simon block 2 (all *ps* > 0.11). The only significant difference for arousal was observed after Game 2 (see Fig. 3), indicating lower arousal under competition game induction (6.44) than after the cooperation induction (7.44) (*p* < 0.05).

### Response times

Incorrect responses (2.5%) and all trials in which RTs less than 150 ms or greater than 1000 ms (Dolk, et al., 2013; Liepelt, et al., 2011; Röder, Kusmierek, Spence, & Schicke, 2007) were excluded for statistical analysis. We submitted RTs to an analysis of variance (ANOVA) with compatibility (compatible, incompatible), and shared experience (playing alone, playing together with a partner) as within-subjects factors and metacontrol state (competition, cooperation) as a between-subjects factor.

**Fig. 4:**
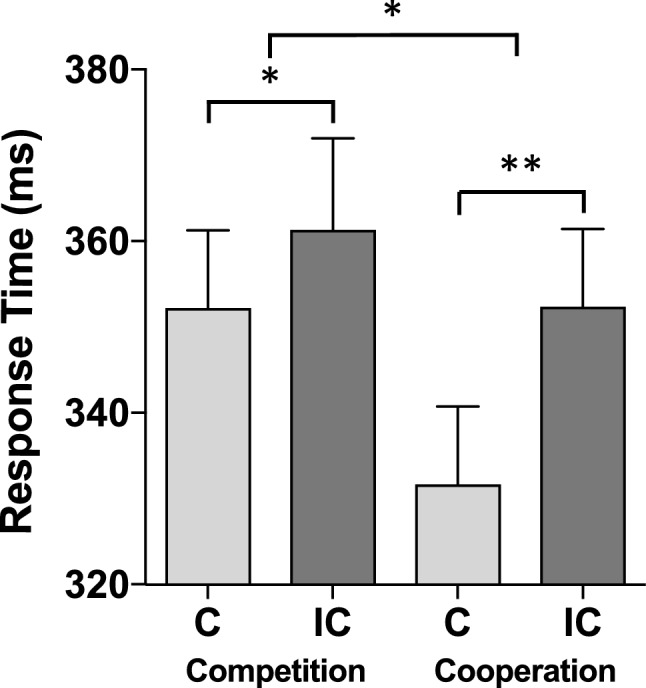
S–R compatibility effect (= JSE) under competition (left side) and cooperation (right side). *C* compatible, *IC* incompatible. Error bars represent standard errors of the mean. *p < 0.05; **p < 0.01

**Fig. 5:**
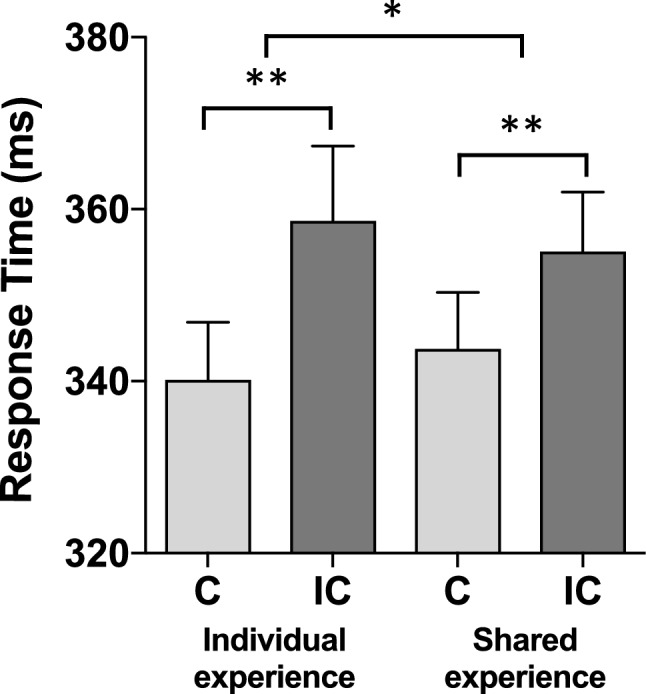
S–R compatibility effect (= JSE) under individual experience (left side) and shared experience (right side). *C* compatible, *IC* incompatible. Error bars represent standard errors of the mean. *p < 0.05; **p < 0.01

The 2 × 2 × 2 ANOVA showed a significant main effect of compatibility, *F*(1, 30) = 30.46, *p* < 0.001, *η*_*p*_^2^ = 0.50, indicating response times to be faster in S–R compatible (mean RT = 342 ms, standard error, SE = 6 ms) than in S-R incompatible conditions (mean RT = 357 ms, SE = 8 ms). Neither the main effect of Metacontrol state, *F*(1, 30) = 1.16, *p* = 0.29, *η*_*p*_^2^ = 0.04, nor the main effect of Shared experience, (*F* < 1), was significant. The interaction of metacontrol state × compatibility (see Fig. 4) was significant, *F*(1, 30) = 4.55, *p* < 0.05, *η*_*p*_^2^ = 0.13, indicating that the S–R compatibility effect was significantly smaller in the competition mode (9 ms) than in the cooperation mode (21 ms). The interaction of shared experience × compatibility (see Fig. 5) was significant, as well, *F*(1, 30) = 4.22, *p* < 0.05, *η*_*p*_^2^ = 0.12, showing that the S–R compatibility effect was significantly smaller when the induction was performed together with a partner (11 ms) than when the induction was performed alone (18 ms). The three-way interaction of compatibility × metacontrol state × shared experience was, however, not significant, *F*(1, 30) = 1.09, *p* = 0.31, *η*_*p*_^2^ = 0.04, indicating that both effects of metacontrol state and shared experience are statistically independent of each other (see Table 1).

**Table 1:** Response times (standard errors SE in brackets) for S–R compatible (C) and S–R incompatible (IC) conditions under cooperation and competition, when induction was performed together (shared experience) and alone (individual experience)

	Cooperation		Competition	
	Shared experience	Individual experience	Shared experience	Individual experience
C	334 (9)	329 (9)	354 (9)	351 (9)
IC	349 (10)	356 (12)	361 (10)	362 (12)

Error rates (see Table 2) were rather low, due to the ease of the joint Simon task. Only the main effect of compatibility was significant, *F*(1, 30) = 12.81, *p* < 0.01, *η*_*p*_^2^ = 0.30, indicating smaller error rates in S–R compatible (1.2%, SE = 0.29) than in S–R incompatible conditions (3.9%, SE = 0.76). All other main effects or interactions were not significant (*F* < 1.93, *p* > 0.17).

**Table 2:** Mean error rates (*M*) and standard errors (SE) for S–R compatible and S–R incompatible conditions under cooperation mode and competition mode, when induction was performed alone and together

	S–R compatible		S–R incompatible	
	*M*	SE	*M*	SE
Cooperation	1.5	0.4	4.8	1.1
Competition	1.0	0.4	2.9	1.1
Alone	1.2	0.3	4.4	1.0
Together	1.3	0.4	3.3	0.6

### Effects of competitive and cooperative state induction and conceptual self-other relation

**Fig. 6:**
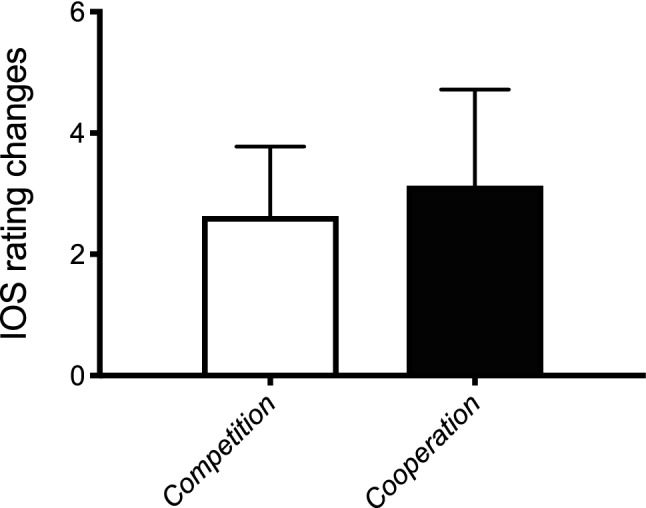
Mean IOS rating changes and standard deviations (error bars) under competition mode and cooperation mode

We submitted the IOS scale data (Aron, et al., 1992) to a Mann–Whitney *U* test with metacontrol state (competition, cooperation) as a between-subjects factor (see Fig. 6). We found no significant differences in the IOS ratings between the competition induction (2.6) and the cooperation induction (3.1) (*p* > 0.36).

## Discussion

The joint Simon effect is considered as a marker for bodily self-other integration in joint action (Colzato, Zech, et al., 2012). This study tested two questions with regard to self-other integration. First, does a competitive processing mode (i.e. metacontrol state) between partners change the degree of self-other integration in joint action? Second, and more exploratory, does competition transfer depend on a shared group experience with a joint action partner?

The data of our manipulation check indicates that the induction of the different processing modes (competition and cooperation) effectively worked via shared game experience. Our results showed that a competitive processing mode produces less bodily self-other integration than a cooperative processing mode (Hommel, et al., 2009; Iani, et al., 2011, 2014) as indexed by a transfer effect to a Joint Simon Task that was performed after the state induction. In line with previous studies (Iani, et al., 2014; Ruissen & de Bruijn, 2016) our findings indicate that the specific metacontrol state a dyadic pair was set to (i.e., competition or cooperation) was transferable to a fully new dyadic task. The transferable metacontrol state can therefore be considered as an abstract control policy acting on a lower level process of bodily self-other integration. The finding that less bodily self-other integration is observed under a competitive processing mode than under a cooperative processing mode (Hommel, et al., 2009; Iani, et al., 2011, 2014) in a consecutively performed joint Simon Task is in line with findings from social psychology (Bargh & Chartrand, 2000; Sassenberg, Moskowitz, Jacoby, & Jansen, 2007). These studies show that participant’s seem to behave in line with a primed mindset even in a new and task unrelated context. Our first question can, therefore, be clearly answered with yes. A competitive processing mode (i.e. metacontrol state) between partners reduces the degree of bodily self-other integration as compared to a cooperative processing mode. Even though the joint Simon effect was significantly reduced following a competitive induction, it was nevertheless present after competition with the same co-actor. This is different to findings of Iani et al. (2014) showing a complete absence of the joint Simon effect after having performed a joint Flanker task in competition. One difference between our study and the study of Iani et al. (2014), which could explain this difference, is the similarity between the induction task and the transfer task applied. The joint Simon task is a spatial compatibility task and the joint Flanker task is non-spatial in nature. However, these two tasks have more similarities than the Mindflex task of our study and the joint Simon task. The joint Simon and the joint Flanker task are both S–R compatibility tasks with overt spatially distributed manual responses (the joint Flanker task containing an additional S–S compatibility effect). Opposed to this, our Mindflex task is much more abstract and mental in nature possessing much less overlap with the joint Simon task. Applied task sets that are activated or deactivated by the respective goal will therefore probably lead to a much more complete “competition transfer” in the study of Iani et al. (2014) than in ours. This speculation could explain why the reduced joint Simon effect following a competitive induction was still significant in our study. Furthermore, based on the findings of Iani et al. (2014), the conclusion that the metacontrol transfer, which we observed in our study, might be better explained with a transfer of competition (not a transfer of cooperation) seems justified.

In contrast to the competition transfer that we observed in the joint Simon task, the competitive state vs. cooperative state induction did not affect the subjective conceptual self-other relation measured with the IOS scale (Aron, et al., 1992). In a recent review (Quintard, Jouffre, Hommel, & Bouquet, 2020), the impact of romantic love and corresponding positive affects due to romantic feelings have been proposed to bias metacontrol states towards flexibility vs. integration also affecting overlapping bodily self-other representations. In line with this proposal, we found evidence for such metacontrol biases on bodily self-other representations (i.e. the JSE). However, the short-term competitive state vs. cooperative state induction that we used to implement different metacontrol state biases, did not affect the conceptual self-other relation between the two co-actors in a way that would be predicted with long-term varying degrees of romantic love and passion felt for the partner as shown in couples (Quintard, Jouffre, Croizet, Bouquet, 2020). This seems to suggest that the degree of bodily self-other representations may be more flexible and easier to change online than conceptual self-other relations, as the latter have been shown to be the product of long-term experiences and long-lasting relations (Quintard, Jouffre, Croizet, et al., 2020). The different degrees of bodily self-other representations varied through different types of metacontrol states, as done in our study, seem to be sensitive to the current online interaction situation. On the other hand, changing degrees of conceptual self-other relation varied through different degrees of passion and romantic love, may be more strongly related to the type of the long-term interactive social relation between persons.

### Alternative emotional accounts

Some studies showed that the degree of self-other integration does increase when participants are in a positive mood state (Kuhbandner, et al., 2010) or when interacting with a friendly interaction partner (Hommel, et al., 2009). It may be possible that decreased bodily self-other integration effects when being in a competitive processing mode than in a cooperative processing mode may be indirect effects of mood changes, since competition may lead to a more negative mood state than cooperation. However, our affect grid data did not show evidence for a decrease in valence under the competition than under the cooperation induction. Therefore, we consider an indirect emotional effect assuming negative mood to be the cause for the observed decrease of bodily self-other integration under competition in our study unlikely. However, we found evidence for lower arousal when being in a competition mode than in a cooperation mode, at least directly after second game induction. Decreasing arousal levels after the competition game might therefore contribute to the observed differences in the size of the JSE found between competitive and cooperative processing modes. The relation between changing metacontrol states and potentially corresponding arousal changes may therefore be worthwhile further investigation.

### Effects of shared experience on self-other integration

Regarding our second question, our exploratory results show for the first time that being together with the partner during the induction phase decreases bodily self-other integration in a consecutive joint Simon task as compared to a situation in which the induction was performed alone. Bodily self-other integration was smaller (and not larger) when the induction was performed jointly, as compared to an individual induction. This finding may be in line with considerations assuming competition transfer and not cooperation transfer (Iani, et al., 2014), as discussed also in the current study. However, as we did not include a neutral baseline condition in our study, this finding might also be explained by the assumption that participants decrease their effort during the joint Simon task after having experienced the induction phase together with the partner and mobilized more effort when having experienced the induction alone. The fear of losing might actually be higher after virtually interacting with a person with whom another task will be shared later on physically. What our findings imply is that the former effect of metacontrol state transfer seems not to depend on a shared group experience, as both effects (metacontrol state and shared experience) seem to be statistically independent of each other. However, this conclusion should be taken with care, as the findings regarding shared experience were more exploratory and our sample size was not too large. Yet, the lack of a statistical three-way interaction seems not to be due to insufficient power as implied by our power simulation showing that not only the number of participants, but also the number of data points seem to be decisive for power estimations. Therefore, we would consider the factors metacontrol state and shared experience, as two separate factors.

The metacontrol states we induced contain a rather abstract competition goal. Thus, a new finding of our study is that abstract cognitive goal states seem to be transferable in socially shared task contexts, an assumption that is in line with the theorizing of different metacontrol state theories (Goschke, 2003; Hommel, 2015). The reason why we think this transfer relates to relatively abstract cognitive goal states is, because we found evidence for transfer between a relatively abstract mentally shared task and a shared action-oriented spatial S-R compatibility task, impacting the degree of bodily self-other integration in joint action.

### A competition mindset affects bodily self-other integration

Our finding of a reduced Simon effect after competitive metacontrol state induction is in line with traditional theories from social psychology, such as the group conflict theory (Campbell, 1965). One assumption of this theory that has been lately stressed is that negative interdependence between different groups leads to prejudice and social discrimination (Iani, et al., 2014; Sassenberg, et al., 2007). Perceiving a conflict is enough to produce effects of prejudice (Esses, Jackson, & Armstrong, 1998) suggesting effects of competition beyond the current situation allowing for possible transfer. The concept of metacontrol has a lot in common with the concept of mindset (Gollwitzer, Heckhausen, & Steller, 1990). Mindset has been defined as a cognitive procedure relevant for choosing between different goal alternatives, producing action planning to attain certain action goals (Gollwitzer, et al., 1990; Sassenberg, et al., 2007). Sassenberg et al. (2007) showed that competition effects can be transferred leading to higher levels of prejudice even in new situations and with new persons that were not involved in actual competition. Our findings are in line with the latter findings also showing that effects of competition do impact social interaction up to the bodily level of self-other integration. Further, our findings suggest that transfer effects of competition can be produced virtually without much bodily social interaction with another person. An interesting line for future research would be to test if the metacontrol state effect we observed is not only transferable to new task situations, but if this effect would even survive a change of the involved co-actor.

### Implications of metacontrol state transfer for theories on joint action

The present findings do not differentiate between the different theoretical accounts (action co-representation and referential coding). But our findings may have implications for both accounts. Assuming that the joint Simon effect is the result of automatic action co-representation (Kiernan, et al., 2012; Sebanz, et al., 2003, 2005), the given findings showing a modulation of the joint Simon effect due to a previously established metacontrol state suggest that co-representation is not fully automatic, but may be context dependent. The independence that is established between two persons by competition affects the amount and strength of co-representation in a new dyadic situation. That competitive or cooperative task goals affect subsequent interaction behavior may be taken as evidence for the strength of social situatedness and the role of social embedding (Barsalou, 2008; Vygotsky, 1978). Referential coding assumes that the joint Simon effect arises from self-other integration of similar action events and the corresponding need to discriminate between them (Dolk, et al., 2013). The degree of actual and perceived similarity between own and others actions determines the strength of the required action discrimination. Taken the given findings and effects of metacontrol on the joint Simon effect in light of referential coding may suggest that setting up abstract competition and cooperation goals affect the amount of perceived similarity of both action partners in a task independent way. Perceiving an interaction partner as less similar, due to a previously induced competitive action goal, seem to reduce the need to discriminate between own and others actions by means of referential coding.

Our findings also have implications for applied joint action situations. For instance, in sports it’s natural that athletes of the same team cooperate in competitions however they as well compete of the very few places in the starting squad or in the national team. The way how coaches may instruct athletes on the competitive and cooperative nature of training and actions may change the way to perceive your teammates and jointly shared action goals.

A limitation of our study is that we cannot clearly distinguish if the given findings are due to less bodily self-other integration under the competition goal or more self-other integration under the cooperation goal, as we had no neutral condition involved. However, as previous work (Iani, et al., 2014; Sassenberg, et al., 2007) have shown clear evidence for the former, and there is evidence that participants typically perceive a “neutral” social task as being cooperatively (Iani, et al., 2011), we think that the assumption of a reduced bodily self-other integration under competition is plausible and in line with the given literature. From these previous studies it becomes evident that finding a good neutral condition is always difficult, as this condition may be reinterpreted positive by the participants in terms of interdependence. Further, due to the relatively small basic joint Simon effect, adding further factors to the design is likely to diminish chances of finding additional interaction effects.

In sum, our findings show the role of abstract cognitive states controlling task features relevant for joint action research providing evidence in favor of metacontrol state theories (Dreisbach, 2006; Goschke, 2003; Hommel, 2015). Our study suggests that metacontrol state theories seem to apply to social dyadic interactions, as well. Understanding if and how shared goals transfer from one task to another may help to foster the understanding of when competition or cooperation is beneficial in consecutively changing social environments and interaction contexts.

## Footnotes

1 We performed an additional analysis adding order of the shared experience (performing the induction alone or together with the partner) to the planned analysis. This analysis showed that there was neither a main effect of order, nor any interaction effects with other factors (all *p*s between > .17 and .95).

## References

[CR1] Aron A, Aron EN, Smollan D (1992). Inclusion of other in the self scale and the structure of interpersonal closeness. Journal of Personality and Social Psychology.

[CR2] Bargh JA, Chartrand TL, Reis HT, Judd CM (2000). The mind in the middle: A practical guide to priming and automaticity research. Handbook of research methods in social and personality psychology.

[CR3] Barsalou LW (2008). Grounded cognition. Annual Review of Psychology.

[CR4] Beringer J (2000). Experimental runtime system (Version 3.33e) [Computer software].

[CR5] Campbell, D. T. (1965). Ethnocentric and other altruistic motives. In D. Levine (Ed.), *Nebraska symposium on motivation, Bd. 13* (pp. 283–311). University of Nebraska Press.

[CR6] Colzato LS, de Bruijn ERA, Hommel B (2012). Up to “me” or up to “us”? The impact of self-construal priming on cognitive self-other integration. Frontiers in Psychology.

[CR7] Colzato LS, van den Wildenberg WP, Hommel B (2013). Increasing self-other integration through divergent thinking. Psychonomic Bulletin and Review.

[CR8] Colzato LS, Zech H, Hommel B, Verdonschot R, van den Wildenberg WPM, Hsieh S (2012). Loving-kindness brings loving-kindness: The impact of Buddhism on cognitive self-other integration. Psychonomic Bulletin and Review.

[CR9] Dittrich K, Dolk T, Rothe-Wulf A, Klauer KC, Prinz W (2013). Keys and seats: Spatial response coding underlying the joint spatial compatibility effect. Attention, Perception, and Psychophysics.

[CR10] Dittrich K, Rothe A, Klauer KC (2012). Increased spatial salience in the social Simon task: A response-coding account of spatial compatibility effects. Attention, Perception and Psychophysics.

[CR11] Dolk T, Hommel B, Colzato LS, Schütz-Bosbach S, Prinz W, Liepelt R (2011). How “social” is the social Simon effect?. Frontiers in Psychology.

[CR12] Dolk T, Hommel B, Colzato LS, Schütz-Bosbach S, Prinz W, Liepelt R (2014). The joint Simon effect: A review and theoretical integration. Frontiers in Psychology.

[CR13] Dolk T, Hommel B, Prinz W, Liepelt R (2013). The (not so) social Simon effect: A referential coding account. Journal of Experimental Psychology: Human Perception and Performance.

[CR14] Dolk T, Prinz W, Cross ES, Obhi SS (2016). What it takes to share a task: Sharing versus shaping task representations. Shared representations: Sensorimotor foundations of social life.

[CR15] Dreisbach G (2006). How positive affect modulates cognitive control: The costs and benefits of reduced maintenance capability. Brain and Cognition.

[CR16] Esses VM, Jackson LM, Armstrong TL (1998). Intergroup competition and attitudes toward immigrants and immigration: An instrumental model of group conflict. Journal of Social Issues.

[CR17] Gollwitzer PM, Heckhausen H, Steller B (1990). Deliberative and implemental mind-sets: Cognitive tuning toward congruous thoughts and information. Journal of Personality and Social Psychology.

[CR18] Goschke T, Maasen S, Prinz W, Roth G (2003). Voluntary action and cognitive control from a cognitive neuroscience perspective. Voluntary action: Brains, minds, and sociality.

[CR19] Hommel B, Andrew JE (2015). Between persistence and flexibility: The Yin and Yang of action control. Advances in Motivation Science.

[CR20] Hommel B, Colzato LS, van den Wildenberg WP (2009). How social are task representations?. Psychological Science.

[CR21] Hommel B, Müsseler J, Aschersleben G, Prinz W (2001). The theory of event coding (TEC): A framework for perception and action planning. Behavioral and Brain Sciences.

[CR22] Hommel B, Wiers RW (2017). Towards a unitary approach to human action control. Trends in Cognitive Sciences.

[CR23] Iani C, Anelli F, Nicoletti R, Arcuri L, Rubichi S (2011). The role of group membership on the modulation of joint action. Experimental Brain Research.

[CR24] Iani C, Anelli F, Nicoletti R, Rubichi S (2014). The carry-over effect of competition in task-sharing: Evidence from the joint Simon task. PLoS ONE.

[CR25] Kiernan D, Ray M, Welsh TN (2012). Inverting the joint Simon effect by intention. Psychonomic Bulletin and Review.

[CR26] Klempova B, Liepelt R (2016). Do you really represent my task? Sequential adaptation effects to unexpected events support referential coding for the joint Simon effect. Psychological Research.

[CR27] Kornblum S, Hasbroucq T, Osman A (1990). Dimensional overlap: Cognitive basis for stimulus–response compatibility—a model and taxonomy. Psychological Review.

[CR28] Kuhbandner C, Pekrun R, Maier MA (2010). The role of positive and negative affect in the “mirroring” of other persons’ actions. Cognition and Emotion.

[CR29] Liepelt R, Prinz W (2011). How two share two tasks: Evidence of a social psychological refractory period effect. Experimental Brain Research.

[CR30] Liepelt R, Schneider JC, Aichert DS, Wöstmann N, Dehning S, Möller H-J, Riedel M, Dolk T, Ettinger U (2012). Action blind: Disturbed self-other integration in schizophrenia. Neuropsychologia.

[CR31] Liepelt R, Wenke D, Fischer R (2013). Effects of feature integration in a hands-crossed version of the social Simon paradigm. Psychological Research Psychologische Forschung.

[CR32] Liepelt R, Wenke D, Fischer R, Prinz W (2011). Trial-to-trial sequential dependencies in a social and non-social Simon task. Psychological Research Psychologische Forschung.

[CR33] Ma K, Hommel B (2018). Metacontrol and body ownership: Divergent thinking increases the virtual hand illusion. Psychological Research.

[CR34] Mendl J, Fröber K, Dolk T (2018). Are You keeping an eye on me? The Influence of competition and cooperation on joint Simon task performance. Frontiers in Psychology.

[CR35] Müller BCN, Brass M, Kühn S, Tsai C-C, Nieuwboer W, Dijksterhuis A, van Baaren RB (2011). When Pinocchio acts like a human, a wooden hand becomes embodied. Action co-representation for non-biological agents. Neuropsychologia.

[CR36] NeuroSky. (2018). Mindflex Duel [Apparatus and software]. Retrieved 15 Nov 2018 from https://store.neurosky.com/products/mindflex-duel

[CR37] Porcu E, Bölling L, Lappe M, Liepelt R (2016). Pointing out mechanisms underlying joint action. Attention, Perception, and Psychophysics.

[CR38] Prinz W (1997). Perception and action planning. European Journal of Cognitive Psychology.

[CR39] Prinz W (2015). Task representation in individual and joint settings. Frontiers in Human Neuroscience.

[CR40] Puffe L, Dittrich K, Klauer KC (2017). The influence of the Japanese waving cat on the joint spatial compatibility effect: A replication and extension of Dolk, Hommel, Prinz, and Liepelt (2013). PLoS ONE.

[CR41] Quintard V, Jouffre S, Croizet JC, Bouquet CA (2020). The influence of passionate love on self-other discrimination during joint action. Psychological Research.

[CR42] Quintard V, Jouffre S, Hommel B, Bouquet CA (2020). Embodied self-other overlap in romantic love: A review and integrative perspective. Psychological Research.

[CR43] Röder B, Kusmierek A, Spence C, Schicke T (2007). Developmental vision determines the reference frame for the multisensory control of action. PNAS.

[CR44] Ruissen MI, de Bruijn ERA (2016). Competitive game play attenuates self-other integration during joint task performance. Frontiers in Psychology.

[CR45] Russel JA, Weiss A, Mendelsohn GA (1989). The affect grid: A single-item scale of pleasure and arousal. Journal of Personality and Social Psychology.

[CR46] Ruys KI, Aarts H (2010). When competition merges people’s behavior: Interdependency activates shared action representations. Journal of Experimental Social Psychology.

[CR47] Sassenberg K, Moskowitz GB, Jacoby J, Hansen N (2007). The carry-over effect of competition: The impact of competition on prejudice towards uninvolved outgroups. Journal of Experimental Social Psychology.

[CR48] Sebanz N, Bekkering H, Knoblich G (2006). Joint action: Bodies and minds moving together. Trends in Cognitive Science.

[CR49] Sebanz N, Knoblich G, Prinz W (2003). Representing others’ actions: Just like one’s own?. Cognition.

[CR50] Sebanz N, Knoblich G, Prinz W (2005). How two share a task: Corepresenting stimulus-response mappings. Journal of Experimental Psychology: Human Perception and Performance.

[CR51] Simon JR (1969). Reactions toward the source of stimulation. Journal of Experimental Psychology: General.

[CR52] Simon JR, Hinrichs JV, Craft JL (1970). Auditory S–R compatibility: Reaction time as a function of ear-hand correspondence and ear-response-location correspondence. Journal of Experimental Psychology.

[CR53] Simon JR, Small AM (1969). Processing auditory information: Interference from an irrelevant cue. Journal of Applied Psychology.

[CR54] Simon JR, Wolf JD (1963). Choice reaction time as a function of angular stimulus-response correspondence and age. Ergonomics.

[CR55] Steiner ID (1972). Group processes and productivity.

[CR56] Stenzel A, Chinellato E, Bou MA, del Pobil AP, Lappe M, Liepelt R (2012). When humanoid robots become human-like interaction partners: Corepresentation of robotic actions. Journal of Experimental Psychology: Human Perception and Performance.

[CR57] Stenzel A, Chinellato E, del Pobil AP, Lappe M, Liepelt R (2013). How deeply do we include robotic agents in the self?. International Journal of Humanoid Robotics.

[CR58] Stenzel A, Liepelt R (2016). Joint Simon effects for non-human co-actors. Attention, Perception, and Psychophysics.

[CR59] Tomasello M, Carpenter M, Call J, Behne T, Moll H (2005). Understanding and sharing intentions: The origins of cultural cognition. The Behavioral and Brain Sciences.

[CR60] Vesper C, Butterfill S, Knoblich G, Sebanz N (2010). A minimal architecture for joint action. Neural Networks.

[CR61] Vygotsky LS (1978). Mind in society: The development of higher psychological processes.

[CR62] Wenke D, Atmaca S, Holländer A, Liepelt R, Baess P, Prinz W (2011). What is shared in joint action? Issues of co-representation, response conflict, and agent identification. Review of Philosophy and Psychology.

[CR63] Westfall, J. (2016). PANGEA (v0.2): Power analysis for general anova designs. [Shiny App]. Retrieved from https://www.jakewestfall.shinyapps.io/pangea/

[CR64] Yamaguchi M, Wall HJ, Hommel B (2017). No evidence for shared representations of task sets in joint task switching. Psychological Research.

[CR65] Yamaguchi M, Wall HJ, Hommel B (2018). Sharing tasks or sharing actions? Evidence from the joint Simon task. Psychological Research.

[CR66] Yamaguchi M, Wall HJ, Hommel B (2019). The roles of action selection and actor selection in joint task settings. Cognition.

